# Anxiety Effect on Communication Skills in Nursing Supervisors: An Observational Study

**DOI:** 10.3390/nursrep11020021

**Published:** 2021-04-01

**Authors:** Ana Colomer-Sánchez, Diego Ayuso-Murillo, Alejandro Lendínez-Mesa, Carlos Ruiz-Nuñez, Guadalupe Fontán-Vinagre, Iván Herrera-Peco

**Affiliations:** 1 Faculty of Communication and Art, University of Nebrija, Calle Hostal s/n, 28240 Madrid, Spain; ana.colomer.universidad@gmail.com; 2 Consejo General de Enfermería, Calle Fuente del Rey 2, 28023 Madrid, Spain; d.ayuso@consejogeneralenfermeria.org (D.A.-M.); g.fontan@ieinstituto.es (G.F.-V.); 3 Nursing Department, Faculty of Health Sciences, Alfonso X el Sabio University, Avda. Universidad 1, Villanueva de la Cañada, 28691 Madrid, Spain; alendmes@uax.es; 4 High Resolution Hospital, APES Poniente, 18300 Granada, Spain; ruiznu.carlosantonio@gmail.com; 5 Alfonso X el Sabio University Foundation, Avda. Universidad 1, Villanueva de la Cañada, 28691 Madrid, Spain

**Keywords:** anxiety, communication, nursing management, personality traits

## Abstract

Communication represents an essential skill in nurse managers’ performance of everyday activities to ensure a good coordination of the team, since it focuses on the transmission of information in an understandable way. At the same time, anxiety is an emotion that can be caused by demanding and stressful work environments, such as those of nurse managers. The aim of the present study was to analyze the impact of anxiety management on nurse managers’ communication skills. The sample comprised 90 nursing supervisors from hospitals in Madrid, Spain; 77.8% were women, and 22.2% were men, with an average of 10.9 years of experience as nursing supervisors. The instruments used for analysis were the Sixteen Personality Factor Questionnaire: version five (16PF5) and State Trait Anxiety Inventory (STAI) questionnaires, validated for the Spanish population. The results showed that emotional stability was negatively affected by anxiety (r = −0.43; *p* = 0.001), while apprehension was positively affected (r = 0.382; *p* = 0.000). Nursing supervisors, as managers, were found to possess a series of personality factors and skills to manage stress and communication situations that prevent them from being influenced by social pressure and the opinion of others.

## 1. Introduction

Nowadays, work processes carried out in hospitals and primary care centers have become more complex because of rapid technological advances and their increased complexity, high workload, and ethical dilemmas, among other factors [1,2]. This leads to situations that cause anxiety, stress, or even depression-related problems [3,4,5]. Nurses’ competencies to interact and communicate both with other health professionals and patients may be diminished because of such anxiety [6], and even communication between professionals may be affected on the management side.

Communication is a dynamic and very complex process that requires the sender of the message to be aware of the barriers and possible limitations that may prevent the information from being conveyed and understood by the receiver of the message [7,8]. Thus, effective communication requires not only mastering the sending, understanding, and monitoring of the information [9], but also properly handling both emotional and social aspects, which requires a high degree of empathy [10] as well as assertiveness and active listening, among other skills [11]. Effective communication in the field of nursing management is an essential tool that facilitates organizational processes, minimizes conflict management that can lead to unhealthy work environments, and increases the quality of care [12,13]. Effective communication of nurse managers with their team members means that they will receive clear information, which generates trust in addition to providing both emotional and work support [14,15].

Anxiety is a normal emotion, and its main purpose is to trigger adaptive behaviors that allow the person to respond to events that can generate tension and stress [1]. However, anxiety is more commonly understood as a complex and unpleasant emotion that is expressed by a feeling of fear and emotional tension, accompanied by an important array of somatic symptoms [16]. If the situation that generates such anxiety extends over time or is of great intensity, it can generate emotional changes that may lead to pathological manifestations in the individual [17].

Situations that generate a state of high stress, anxiety, and even depression in nurses lead to both physical and psychological exhaustion [18]. Moreover, they negatively influence nurses’ perception of others’ needs, rendering them less capable of understanding them and generating “compassion fatigue” [19,20], diminish nurses’ adaptability to change [1], and reduce and hinder their ability to convey information in an efficient manner [21,22,23,24,25,26].

Nursing supervisors, in their position as ward managers, play an essential role in the direct management of nurses’ duties, and are thus one of the key elements supporting the well-being of the work environment, the management of shifts, and the quality and safety of the care offered by their team, amongst other functions [27,28]. The effect of stress-generating situations also affects communication skills, resulting in a bad organizational climate [18,29]. This can generate hostility toward the work environment and authoritarian behaviors that can even derive in humiliating treatment of nurses by their superiors, causing a loss of confidence among professionals, with a significant impact on communication [30,31].

Based on the theoretical proposals above, we observed a lack of evidence in relation to the analysis of anxiety and communication in nurse managers; hence, this research aimed to analyze the role of personality traits in the management of anxiety and their potential link to communication-related personality traits in nurse managers. The main aim of this study was to explore the possible relation between interpersonal communication abilities and anxiety-management skills. With regard to this objective, the following research hypotheses were posed:

**Hypothesis** **1.** *Nursing supervisors, as managers, have communication-related personality traits above the population average.*

**Hypothesis** **2.** *Nursing supervisors have greater resistance to stress-generating situations than the population average.*

**Hypothesis** **3.** *It is expected that anxiety could have an association with some communication components.*

## 2. Materials and Methods

### 2.1. Participants and Sampling

This study focused on nurses who have managerial duties in hospitals belonging to the Servicio Madrileño de Salud (Madrid Health Service–MHS), in Madrid, Spain. We obtained a total of 90 completed questionnaires from nurse managers participants. The rate of response to our study was 9.31%, as the total number of nurse managers in Madrid Health Service was composed of 967 nurses [32].

Therefore, the final sample consisted of 90 participants with an average age of 48.31 years (SD 8.06), ranging from 32 to 61 years. The average experience as a nurse was 26.5 years (SD 8.08), and that as a manager nurse was 9.82 years (SD 6.97). Regarding the distribution by gender, it was observed that 77.8% were women (*n* = 70), and 22.2% (*n* = 20) were men. The mean age of female nurse managers was 49.85 years (SD 7.4), while their experience as nurses was 27.73 years (SD 7.4), and their management experience was 10.9 years (SD 7). In the case of men, it was observed that the mean age was 42.9 years (SD 7.6), their experience as nurses was 20.2 years (SD 7.7), and their management experience was 6.05 years (SD 5.4). The sample characteristics distribution is shown in Table 1.

The inclusion criteria were being nursing supervisors, and having their position assigned to a Hospital Unit.

The process of selecting participants was carried out in the following way. First, the nursing directorates of MHS hospitals were contacted for the recruitment of participants, and they asked to disseminate the questionnaire amongst the nurse managers of each hospital, after informing them about the study and finding out their willingness to participate. Finally, the questionnaire was implemented on a web platform, Google forms, to facilitate its completion online.

### 2.2. Instrument

In the present study, the researchers prepared a questionnaire, designed in two sections, to collect participants’ information. The first was the socio-demographic section, which ask participants about gender, age, and length of experience as a nurse and as a nurse supervisor. The second was the psychometric section, which included the Sixteen Personality Factor Questionnaire: version five (16PF5) and the State Trait Anxiety Inventory (STAI) to assess communication skills and anxiety in participants, respectively.

We used the Spanish adaptation of 16PF5 [33]. This instrument comprises 185 items organized into16 scales. The 16PF5 analyses personality factors to assess and understand the personality of the subject. This questionnaire was derived from the original 16PF questionnaire published by Catell in 1946 [34], and was validated for the Spanish population by Seisdedos in 1990 [33], showing an internal consistency for the 16 factors in a range between 0.54 and 0.84, and an acceptable internal consistency, with a Cronbach’s alpha of 0.75 [35]. The personality traits defined in 16PF5 are divided into primary and global factors, and can be used to measure communication, which is defined by warmth (A), abstract reasoning (B), emotional stability (C), dominance (E), rule-consciousness (G), socially bold (H), sensitivity (I), privacy (N), apprehension (O), openness to change (Q1), and tension (Q4) [33,34].

The mean score for personality traits in the Spanish population that define communication was measured and defined by Seisdedos [33]. The mean score was defined as 5.5 points; values above this value were expressed as (+), and less than 5.5 were expressed as (−).

The State Trait Anxiety Inventory (STAI) was used to assess anxiety. The original questionnaire was developed by Spielberger et al. (1983) [36] and was adapted to the Spanish population by Buela-Casal et al. in 2011 [37]. This tool is made up of 40 items organized in two scales, which allow both state anxiety and trait anxiety to be measured. State anxiety is defined as a transitory emotional condition derived from the situations lived in a specific moment, while trait anxiety is defined as a habitual propensity associated with personality traits that allow the individual to act in stressful and anxiety-inducing situations. The items are scored on a 4-point Likert-type scale. The adaptation of the STAI to the Spanish population showed good internal consistency in terms of the average of inter-item correlations for both state anxiety (Cronbach’s alpha = 0.9) and trait anxiety (Cronbach’s alpha = 0.94) [37]. In the present study, we evaluated trait anxiety. The mean value of the anxiety score in the adult Spanish population was set at 5.5 points by Buela-Casal et al. (2011) after an analysis of state and trait anxiety in 1,500 participants [37]. It is important to establish that there was no significant bias due to gender of participants, and a subsequent analysis of the STAI showed that the trait anxiety subscale has no significant difference in mean score from the 5.5 value and the internal consistency given by Buela-Casal et al. in 2011 [38].

### 2.3. Procedure and Data Analysis

The present research was designed as a quantitative, observational, and cross-sectional study. For data analysis, descriptive and inferential statistics were calculated with the Statistical Package for the Social Sciences software (SPSS) version 23.0 (IBM, Armonk, NY, USA). Descriptive statistics of both communication-related personality traits and anxiety were presented. The Kolmogorov-Smirnov test was used to assess the normality of the data obtained. Regarding the correlational analysis, Spearman’s nonparametric correlation coefficient (Spearman’s Rho) was applied to contrast hypotheses and estimate the strength of the evidence in favor of the alternative hypothesis versus the null hypothesis. The statistical significance level was set at *p* < 0.05.

### 2.4. Ethical Considerations

The research proposal was previously evaluated and approved by the Ethical Clinical Research Committee of the University Hospital of Getafe of Madrid, Spain, the workplace of the first researcher (Reference A11–15). All participants received an invitation letter, additional information about the study, and a request to sign the informed consent form, included in the online questionnaire, if volunteering to participate. Data were collected in November of 2018.

## 3. Results

### 3.1. Assessment of Personality Traits for Communication and Anxiety

The analysis of participants’ communication-related personality traits showed that only one of the traits was below the population mean, namely emotional stability (5.21). All other values were above the population mean. Regarding anxiety, it presented a value of 3.52 (SD 1.61) (Table 2).

The possible effects of socio-demographic variables, namely gender, age, experience as a nurse, and experience as a nurse manager, on the components of communication and anxiety were analyzed. As shown in Table 3, there was a significant correlation of sensitivity with gender (women) (r = −0.377; *p* < 0.000), age (<50) (r = 0.274; *p* < 0.009), and experience as a nurse (>25 years) (r = 0.294; *p* < 0.005) (Table 3).

### 3.2. Descriptive and Correlational Analyses of Anxiety and Communication

Table 4 shows the relationship observed between all communication components and anxiety in the sample of nurse managers analyzed. It can be seen that anxiety was correlated with emotional stability and apprehension. We can observe that emotional stability (r = −0.43; *p* = 0.001) was inversely correlated with anxiety, while apprehension (r = 0.382; *p* = 0.000) had a positive relationship with anxiety.

No relationship was observed between anxiety and the other personality factors analyzed (Table 4).

## 4. Discussion

The empirical study presented here met the aims originally posed, to determine the relational role of anxiety over the different communications components.

Nurse managers may experience problems when they have to communicate with the nursing staff in a clinical context involving situations that generate stress and anxiety. Therefore, this empirical study fulfilled the previously-stated objective of determining the effect of anxiety on the different components of communication.

First, regarding communication-related personality traits, the results showed that in the case of nurse managers, those traits were above the Spanish population average [33]. This matches the results of other studies in which it was observed that these professionals possess high communication skills [38]. The nurse managers included in our study showed values typical of individuals with strong communication skills both socially and emotionally, and who possess a great command of procedures, particularly, in terms of their organizational capacity, conflict management, and flexibility when facing and dealing with complex situations [10,13,15,39]. They proved to have a great command of communication, focused on expressiveness and social control, which are essential when managing and coordinating teams; this was in agreement with previous studies on the communication characteristics of nurse managers [14,39,40]. These findings support our first hypothesis that nurse managers have high scores on communication-related personality traits than the general population.

Second, an important result was obtained in this study, which is that anxiety measured within the nursing supervisor sample, as nursing managers closer to clinical activity, was below the average of the Spanish population (5.5) [37]. This result is similar to others previously published that described nurse managers as having a greater ability to manage stressful environments and situations, allowing them to perform their team management activities [28]. This fact is linked to the acquisition of anxiety-management tools [40,41], as well as to team support and work procedures control [27]. However, it should be noted that despite having a lower level of anxiety than the average of the population, this result was not inconsistent with other studies that showed high levels of anxiety among nurse managers when facing different situations in their everyday practice [1,42,43], which can lead to changes in their mental health status that may affect their team management and coordination activities [38,44].

Third, the possible effect of socio-demographic variables on anxiety and the measured personality factors was analyzed, finding that there was no statistically significant relationship of these variables with anxiety or personality factors. There was only one exception: sensitivity was to be correlated with gender (women), age (<50 years), and experience as a nurse (>20 years). With regards to the gender variable, it is important to note that nurses are a group made up almost exclusively of women, representing 84.2% of the total group, with female gender being associated with characteristics that allow women to be identified as sensitive to the situation of others [1]. Regarding age and experience, it is important to indicate that they were associated with self-reliance and empathy, which, in turn, were closely related to sensitivity towards others, an important characteristic in the humanization of care, which nurses apply constantly [45].

Fourth, the possible effect of poor anxiety-management skills, translated into a high score on anxiety, on communication-related personality traits was analyzed, and it was found that there was a relationship between anxiety and emotional stability, apprehension, and tension. No effect of anxiety was found on the rest of personality traits. We observed that the relationship of anxiety with apprehension and tension indicates that nurse managers, when faced with situations that generate a high level of anxiety, have the ability to manage such situations and prevent their social communication skills from being negatively affected. Both apprehension and tension are associated with the relationship between contact and exchange of ideas and comments with other people [46], that is, they are related to social anxiety, which leads to nurse managers feeling pressure before a possible scrutiny by other people of their behavior and actions associated with their work team management [47,48]. This may be associated with a decrease in their ability to communicate [21,25], as they do not want to expose themselves to others because they fear being wrong or being judged [49]. This situation did not occur in our sample, as we observed that nurse managers clearly did not allow themselves to be influenced by others’ opinions and kept performing their duties to guarantee the adequate management of their teams.

Furthermore, the results showed an inverse relationship between anxiety and emotional stability; thus, nurse managers are realistic and emotionally stable individuals, which means they can perform their team management duties staying calm at all times under stressful situations [27]. One possible explanation for this is the great decision-making capacity and experience of nurse managers [18], since the subjects in our sample had 27.73 (SD 7.4) years of experience as nurses, and 10.9 (SD 7) years as nurse managers. In this sense, it is important to consider the association between experience and self-confidence to satisfactorily address situations of a clinical and managerial nature within nursing work groups [18].

The present study has some limitations, the first of which is related to its design, since this was an observational and cross-sectional study, where a causal relationship between the variables cannot be established. Furthermore, our study was based on self-reported measures to assess personality traits associated with communication and the anxiety perceived by the individual. Finally, another limitations is the gender distribution of the sample, which could be due to the sampling procedure; however, keeping in mind that the Spanish nurse population in is predominantly female, it may be considered representative.

## 5. Conclusions

In the present study, the relationship between personality traits involved in anxiety-inducing situations and those associated with communication skills was explored. The findings of our study suggest that stressful situations can affect elements associated with communication from a social and emotional point of view, such as apprehension, tension, and emotional stability, thus altering nurse managers’ ability to transmit information.

Nurse managers were found to possess a series of personality factors and skills to manage stress and communication situations that allow them to avoid being influenced by social pressure and the opinions of others. Thus, nurse managers, also supported by self-confidence and professional experience, will perform their management work, communicating adequately and precisely to coordinate their team of professionals and work environments.

This study has important implications for the field of nursing team management, since it represents an advance in the knowledge of how anxiety can affect nurse managers in relation to their ability to communicate with other professionals in their charge. This effect on communication skills may entail a huge problem for performing effective management, both when it comes to managing and coordinating work teams themselves, and also regarding logistic and economic aspects in the management of healthcare organizations.

We believe it is important to highlight that communication skills, besides being initially defined by our personality factors, can be improved through training programs and specific interventions, so as to prevent nurse managers’ abilities from being dwindled when they face situations involving significant stress and anxiety.

Finally, as a future line of research, we are focused on developing and implementing an intervention focused both on the improvement of communication skills and on anxiety and stress management by nursing managers. In training programs to promote emotional well-being through managing anxiety and communication, the following initiatives have proven effective in reducing mild mental disturbances in situations with a high emotional impact and should thus be developed and implemented: mindfulness, interventions focused on improving soft skills, role-play game to practice communication skills in different scenarios, or even setting up telephone hotlines to support healthcare professionals, among others initiatives. 

## Figures and Tables

**Table 1:** Sample characteristics.

Variables	*n*	%
Gender		
Female	70	77.8
Male	20	22.2
Age (years)		
<50	52	57.88
>50	38	42.2
Experience as nursing supervisor (years)		
<10	50	55.6
>10	40	44.4
Experience as a nurse (years)		
<25	26	28.9
>25	64	71.1

**Table 2:** Personality traits for communication and anxiety.

Personality Traits	Mean	SD
Warmth	5.73	1.53
Abstract reasoning	5.92	1.29
Emotional stability	5.21	1.13
Dominance	6.08	1.65
Rule-consciousness	5.53	1.53
Socially bold	5.8	1.68
Sensitivity	6.36	1.85
Privacy	6.06	1.77
Apprehension	6.12	1.62
Open to change	5.72	1.41
Tension	6.28	1.38
Anxiety	3.52	1.61

SD = standard deviation.

**Table 3:** Relationship between socio-demographic variables, communication components, and anxiety.

Personality Traits	Gender	Age	Exp.NurSup.	Exp.Nur.
	*Rho Spearman (p-Value)*			
Warmth	−0.067 (0.533)	0.021 (0.845)	0.125 (0.24)	−0.033 (0.755)
Abstract reasoning	0.023 (0.828)	−0.144 (0.176)	−0.067 (0.533)	−0.131 (0.219)
Emotional stability	0.204 (0.054)	−0.15 (0.159	−0.58 (0.138)	−0.198 (0.062)
Dominance	0.065 (0.545)	−0.04 (0.711)	0.068 (0.523)	−0.045 (0.672)
Rule-consciousness	0.015 (0.88)	0.001 (0.992)	0.044 (0.679)	−0.034 (0.75)
Socially bold	0.054 (0.613)	−0.015 (0.889)	0.028 (0.796)	−0.017 (0.876)
Sensitivity	−0.377 (0.000) *	0.274 (0.009) *	0.147 (0.166)	0.294 (0.005) *
Privacy	0.065 (0.542)	−0.052 (0.628)	−0.086 (0.422)	−0.88 (0.409)
Apprehension	−0.169 (0.111)	0.034 (0.747)	0.155 (0.145)	0.109 (0.305)
Open to change	−0.014 (0.894)	0.13 (0.224)	0.159 (0.135)	0.116 (0.275)
Tension	−0.006 (0.952)	−0.041 (0.703)	0.021 (0.841)	0.016 (0.883)
Anxiety	−0.011 (0.918)	−0.072 (0.5)	0.088 (0.408)	−0.083 (0.436)

Exp.NurSup. = experience as nursing supervisor with management role (years); Exp.Nur. = experience as nurse (years); * *p* < 0.05.

**Table 4:** Correlation pairs between anxiety and communication items.

Pairs		*r*	Significance	95% CI	
				Lower	Upper
Anxiety	Warmth	−0.41	n.s. (0.686)	−0.23	0.15
	Abstract reasoning	−0.5	n.s. (0.619)	−0.21	0.12
	Emotional stability	−4.09	***	−0.4	−0.14
	Dominance	−0.11	n.s. (0.912)	−0.23	0.21
	Rule-consciousness	0.83	n.s. (0.406)	−0.11	0.27
	Socially bold	−0.27	n.s. (0.788)	−0.24	0.18
	Sensitivity	0.96	n.s. (0.339)	−0.12	0.34
	Privacy	−0.08	n.s. (0.938)	−0.24	0.22
	Apprehension	4.47	***	0.23	0.6
	Open to change	−0.9	n.s. (0.368)	−0.25	0.09
	Tension	3.61	***	0.14	0.47

n.s. = no signifcant; *** *p* < 0.001.

## Data Availability

Not applicable.
